# Long-Term Usage and Improved Clinical Outcomes with Adoption of a COPD Digital Support Service: Key Findings from the RECEIVER Trial

**DOI:** 10.2147/COPD.S409116

**Published:** 2023-06-22

**Authors:** Anna Taylor, Andrew Cushing, Morgan Dow, Jacqueline Anderson, Grace McDowell, Stephanie Lua, Maureen Manthe, Sandosh Padmanabhan, Shane Burns, Paul McGinness, David J Lowe, Christopher Carlin

**Affiliations:** 1 Departments of Respiratory and Emergency Medicine, Queen Elizabeth University Hospital, NHS Greater Glasgow & Clyde, Glasgow, Scotland; 2 Lenus Health Ltd, Edinburgh, Scotland; 3 Institute of Cardiovascular and Medical Sciences, University of Glasgow, Glasgow, Scotland; 4 Institute of Health and Wellbeing, University of Glasgow, Glasgow, Scotland

**Keywords:** COPD, self-management, remote monitoring, patient-reported outcomes

## Abstract

**Purpose:**

Digital tools may improve chronic obstructive pulmonary disease (COPD) management, but further evidence of significant, persisting benefits are required. The RECEIVER trial was devised to evaluate the Lenus COPD support service by determining if people with severe COPD would continue to utilize the co-designed patient web application throughout study follow-up and to explore the impact of this digital service on clinical outcomes with its adoption alongside routine care.

**Patients and Methods:**

The prospective observational cohort hybrid implementation-effectiveness study began in September 2019 and included 83 participants. Recruitment stopped in March 2020 due to COVID-19, but follow-up continued as planned. A contemporary matched control cohort was identified to compare participant clinical outcomes with and minimize biases associated with wider COVID-19 impacts. Utilization was determined by daily COPD assessment test (CAT) completion through the application. Survival metrics and post-index date changes in annual hospitalizations were compared between the RECEIVER and control cohorts. Longitudinal quality of life and symptom burden data and community-managed exacerbation events were also captured through the application.

**Results:**

High and sustained application utilization was noted across the RECEIVER cohort with a mean follow-up of 78 weeks (64/83 participants completed at least one CAT entry on ≥50% of possible follow-up weeks). Subgroup analysis of participants resident in more socioeconomically deprived postcode areas revealed equivalent utilization. Median time to death or a COPD or respiratory-related admission was higher in the RECEIVER cohort compared to control (335 days vs 155 days). Mean reduction in annual occupied bed days was 8.12 days vs 3.38 days in the control cohort. Quality of life and symptom burden remained stable despite the progressive nature of COPD.

**Conclusion:**

The sustained utilization of the co-designed patient application and improvements in participant outcomes observed in the RECEIVER trial support scale-up implementation with continued evaluation of this digital service.

## Plain Language Summary

Why was this Study Done?

- COPD accounts for many hospital admissions and deaths and is expensive to manage.
- Digital tools could improve COPD management but more evidence is needed that people with COPD continue to use them and that they have a positive impact on clinical outcomes.
- The goal of this study was to record usage of a digital COPD self-management and remote monitoring service by people with severe COPD over a long-term period, and to explore the impact of introducing this service on participant’s outcomes.

What Did the Researchers Do/Find?

- The majority of trial participants frequently used the service across the time period that they were in the study (12–24 months).
- Those registered with the service had a greater reduction in hospital admissions related to their COPD than a comparator cohort with severe COPD over the same period.
- Participants reported consistent quality of life across the study, despite COPD being a progressive disease.

What Do these Results Mean?

- People with severe COPD continue to use the service regularly.
- Adoption of the service was associated with improved clinical outcomes.

## Introduction

Chronic obstructive pulmonary disease (COPD) is a leading cause of global mortality, morbidity, and disability,^1^ with increased prevalence in communities with lower socioeconomic status worldwide.^2^,^3^ In the UK, COPD poses a major public health concern with over 1.2 million people estimated to have a diagnosis and 30,000 deaths per year accounted for by COPD.^4^ Delays in diagnosis and inconsistent delivery of optimized guideline-based COPD care contribute to adverse outcomes.^5^ COPD is also a significant driver of UK healthcare expenditure with costs incurred primarily by hospital admissions.^6^ The direct costs of managing COPD to the NHS are projected to increase substantially to £2.5bn per year by 2030 due to increases in incidences of COPD.^7^

Interventions that support self-management of COPD have been shown to reduce length and frequency of hospital admissions,^8^,^9^ reduce mortality,^10^,^11^ improve health-related quality of life,^8^,^12^ and be cost-effective.^13^ However, clinical teams often lack the capacity to provide sufficient support for them to be effective.^14^,^15^ Digital self-management services offer the opportunity to provide scalable access to resources both to replace existing self-management programs and to supplement standard treatment.^15^ As a result, a wide range of digital COPD self-management interventions with different use cases have been developed.^16–20^ Positive results have been seen from individual studies using internet/application-based digital self-management interventions for COPD.^16–18^ However, small sample sizes, heterogeneity of interventions, limited follow-up, and a lack of consistency in both study endpoints and control comparators mean that there is currently insufficient evidence of significant, persisting benefit from these tools.^21^

In parallel, emerging innovations such as smart inhalers, remotely monitored home non-invasive ventilation (NIV) machines, wearable physiology monitors, and predictive AI-models offer potential COPD care-quality improvements. The projected benefits are based on these digitally enabled tools providing actionable insight to clinical care teams in real-time outside of traditional care settings.^22–24^ However, infrastructure is required to embed and further evaluate these tools within current treatment pathways. Advances in cloud-based computing provide enhanced data sharing and structuring capabilities to create this infrastructure and establish the clinical value of these tools.^25^

Given the above context, a digital health technology catalyst project was commenced to further the evidence base for digital self-management and remote monitoring interventions in the management of COPD, and to establish a test-bed infrastructure for additional innovations. To address these aims, a patient and clinician co-designed COPD digital support service (Lenus COPD) was developed and evaluated. This digital COPD support service consists of a patient application, a clinician application/dashboard, and a support website. Additional information on the digital service is available at: https://support.nhscopd.scot.

The RECEIVER (remote management of COPD: evaluating the implementation of digital innovations to enable routine care) trial was commenced in September 2019 in NHS Greater Glasgow and Clyde (NHS GG&C). The aim of the trial was to determine the acceptability, feasibility, and utility of deploying the co-developed COPD digital service alongside routine clinical care for people with severe COPD. An implementation-effectiveness observational cohort design was applied to facilitate adaptations to the intervention and the implementation strategy if required. The primary objective of the study was to determine service utilization by people with severe COPD, with additional clinical and non-clinical secondary objectives outlined in the published protocol.^26^ Trial recruitment was planned between September 2019 and August 2020. A data censor date of the 31st of August 2021 was set to allow for 12–24-months of follow-up.

At the first UK COVID-19 lockdown (March 2020), recruitment for the trial was stopped. However, based on positive interim evaluations, follow-up of the 83 already recruited participants was continued. Based on these same evaluations, provision of the COPD digital service was scaled up to a wider cohort of vulnerable people with COPD resident in NHS GG&C outside of the trial, to mitigate care disruptions for these individuals. The service evaluation analyses from this cohort will be reported separately. Additionally, a location and time-period matched control cohort with severe COPD was identified from an approved de-identified dataset from the NHS GG&C Safe Haven.^27^ This allowed for the potential impact of COVID-19 protective measures on the clinical outcomes of populations with COPD to be accounted for in the subsequent outcome analysis.

Having reached the follow-up censor date for the RECEIVER trial, the primary and core secondary outcomes of the study can now be reported on. These include measures evaluating patient application usage, clinical outcome measures compared to the matched control cohort, characterization of trends in quality of life and symptom burden following service onboarding, and determining community-managed exacerbation count amongst the participants. Results from exploratory analysis to identify potential factors determining utilization and analysis to establish the extent of utilization amongst a subpopulation of participants resident in more deprived postcode areas are also reported on.

## Materials and Methods

### Study Design

The RECEIVER trial is a prospective observational cohort hybrid implementation-effectiveness study, performed according to the UK Policy Framework for Health and Social Care Research.^28^ All recruited participants provided written informed consent. The study was registered prospectively (NCT04240353) and the protocol is published.^26^

### Participants

People with COPD attending secondary care in NHS GG&C were screened for eligibility to be included in the RECEIVER cohort.

RECEIVER cohort inclusion criteria:

- At least 18 years of age at onboarding
- Confirmed diagnosis of COPD (GOLD 2019 criteria)
- A COPD exacerbation requiring hospitalization in the previous 12 months and/or chronic hypercapnic respiratory failure or sleep-disordered breathing meeting established criteria for home NIV/continuous positive airway pressure (CPAP) treatment
- Daily access to a smartphone, tablet, or desktop computer with internet access either personally or through a close contact

RECEIVER cohort exclusion criteria:

- Lack of capacity to give informed consent or having a communication barrier precluding the use of the service

### Intervention

The service consists of:

- A patient web application accessible via smartphone, tablet, or computer where patient-reported outcomes (PROs) can be entered, and standardized self-management advice can be accessed
- A clinician dashboard where structured data from electronic health records (EHRs), PROs, wearables, and home NIV machines are displayed
- An asynchronous messaging facility accessible via the patient application and clinician dashboard which facilitates out of hospital, non-urgent patient-clinician contact
- A support website providing further self-management resources

**Figure 1:**
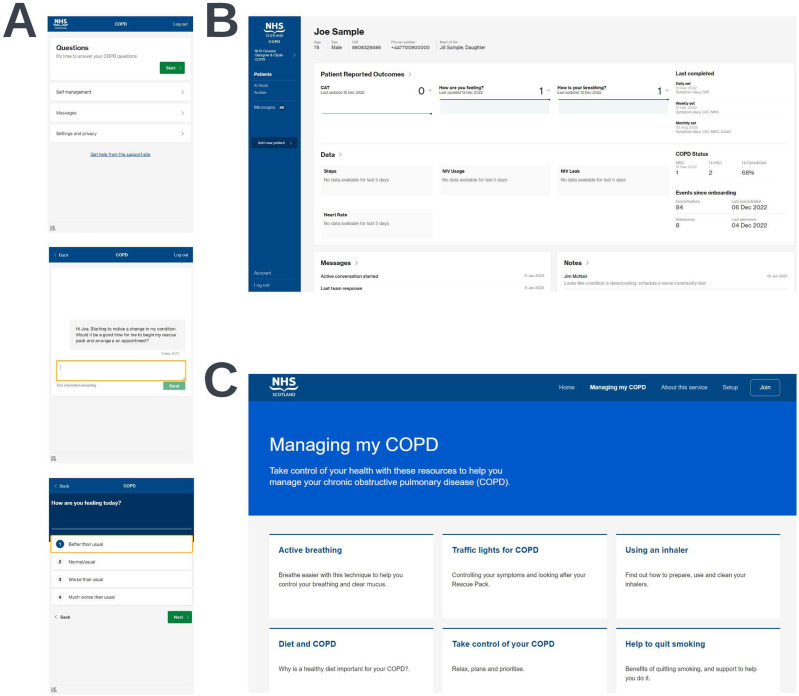
Screen capture views of the three components of the digital COPD support service. Views of the patient application (**A**), the clinician dashboard (**B**), and the support website (**C**) are shown. Synthetic data shown is for illustrative purposes only.

Screen capture views of the intervention components are shown in Figure 1.

The clinician dashboard was utilized to maximize information available and support clinician decision-making following participant-initiated contact through the messaging service or during routine clinical contact (eg, virtual, telephone, or face-to-face appointments). Participants were made aware that their symptom data would not be routinely reviewed or monitored outside of this context and that they should seek medical advice through usual care channels if they felt unwell. Further details of the intervention components and how they were used, as well as details on data processing and data storage are available in the Supplementary Methods.

### Patient and Public Involvement

Semi-structured interviews with people living with COPD in NHS GG&C were undertaken as part of the design of the Lenus COPD digital tools to understand the experience of people living with COPD and to allow for iterative co-design of the COPD digital service. The digital service and the RECEIVER study design were informed by these interactions, alongside published data on the priorities of people with COPD.^29^ Testing early in the study period showed a system usability scale score^30^ of 85/100, which is considered above average for usability and learnability.

### Data Collection

Study follow-up took place from the 3rd of September 2019 to the 31st of August 2021. Recruitment ceased for the RECEIVER cohort in March 2020 due to UK COVID-19 restrictions. PRO data was collected via the patient application, and baseline demographic and physiology data were obtained from EHRs, with all data aggregated into the clinician dashboard. Where available, summary home NIV (ResMed Lumis 150 ST-A) and wearable data (Fitbit Charge 3) were also included in the clinician dashboard. However, analysis of this physiology and therapy data is outside of the scope of this paper.

### Control Cohort Identification Process

The control cohort was established from a linked and deidentified dataset produced by the NHS GG&C Safe Haven^27^ which included demographic, hospital admission, and mortality data for individuals resident in NHS GG&C with COPD. Control cohort identification was carried out by iteratively identifying the individuals within the Safe Haven COPD dataset who met the matching criteria for each RECEIVER participant and then selecting the top five closest matches by age for that participant from this matched group.

These matching criteria were as follows:

- Had a COPD or respiratory-related admission in the seven-days up to the onboarding date of the RECEIVER participant
- Alive at the onboarding date of the RECEIVER participant
- Same sex as the RECEIVER participant
- Not already matched to another RECEIVER participant
- Not a user of the intervention (COPD digital service)

Due to data availability and constraints within the Safe Haven deidentified dataset, it was not possible for additional disease-related criteria to be matched for (eg, medications, co-morbidities, lung function, smoking status). Each RECEIVER participant was matched with five controls to mitigate any biases resulting from the lack of complete data for the control cohort. Alternative time windows of up to 12 months for the preceding COPD/respiratory-related admission for control subjects were considered. As this would have potentially introduced additional biases (eg, seasonal variability of COPD events), and as evaluation would combine 12-month pre-post index event and survival data, a 7-day window was considered to be most appropriate.

### Primary and Secondary Outcome Measures

The primary outcome measure for the trial was the proportion of trial participants utilizing the patient application as determined by completion of daily COPD assessment test (CAT)^31^ entries. Analysis of utilization patterns, subgroup analysis of utilization amongst participants resident in more deprived postcode areas, and exploratory analysis looking at potential factors associated with utilization were also conducted.

Clinical secondary outcomes were directly compared between the RECEIVER and control cohorts with the index date for both the RECEIVER participants and their matched controls set as the RECEIVER participant’s onboarding date. Survival metrics and changes in annual COPD or respiratory-related admissions and occupied bed days were compared between the cohorts. Other secondary outcomes included analysis of changes in health-related quality of life (EQ-5D visual analogue scale)^32^ and symptom burden (CAT score) over the study duration and community-managed exacerbation count during the first year of follow-up. Definitions for COPD or respiratory-related admission and exacerbation events are noted in the Supplementary Methods. Supplementary Table 1 details the outcomes that are reported in this paper, as well as the data that were collected throughout the RECEIVER trial that are not included in the analyses presented in this paper but will be reported on subsequently.

### Statistical Analysis

Utilization at each timepoint was calculated from the participants who were alive and onboarded at that time. Survival analyses were conducted comparing time to admission, death, and admission or death between the RECEIVER, and control cohorts using Kaplan–Meier survival analysis with a Log rank test. Survival between cohorts was compared with median time to event metrics and unadjusted hazard ratios. Wilcoxon signed-rank test effect sizes were used to compare the change in annual admission and occupied bed day (OBD) counts between the cohorts. Kruskal–Wallis tests and chi-squared tests were used as appropriate to explore differences in baseline characteristics and admission events over the first year of follow-up between groups of participants with different levels of application utilization. All statistical analyses were performed using R version 4.0.5.

## Results

### Trial Participant and Control Cohort Flow Diagrams

**Figure 2:**
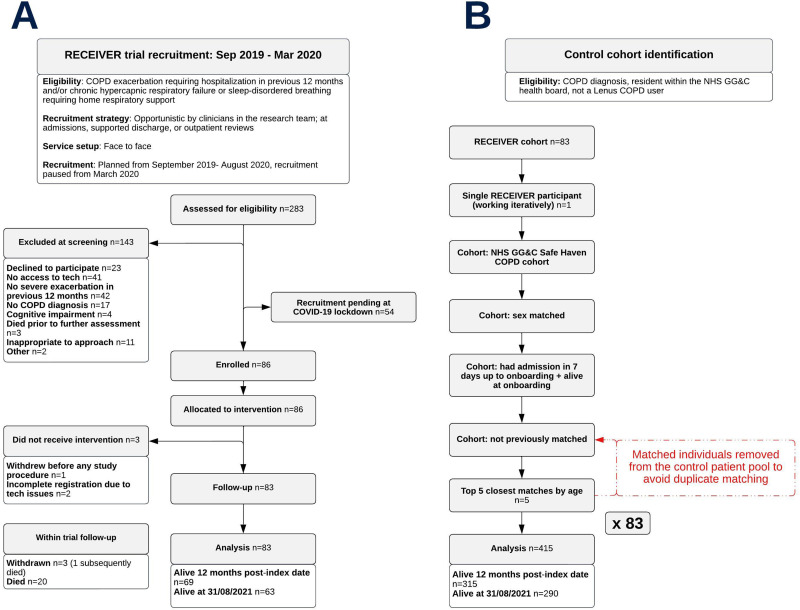
Participant flow diagram for the RECEIVER cohort (**A**) and cohort identification flow diagram for the location and time-period matched control cohort (**B**).

The RECEIVER participant flow diagram details the recruitment strategies for the trial participants (Figure 2A). The control cohort flow diagram details how the control cohort was selected from the Safe Haven COPD dataset (Figure 2B). Twenty individuals in the RECEIVER cohort and 125 individuals in the control cohort died prior to the data censor date. Three individuals in the RECEIVER cohort withdrew from the study, discontinuing PRO completion. They did not have further study procedures but continued with routine clinical follow up including clinical event capture, as per protocol. Accordingly, matched patients were not withdrawn from the control cohort.

### Trial Participant and Control Cohort Characteristics

RECEIVER participant and control cohort characteristics are summarized in Table 1. The RECEIVER and control cohorts were well matched based on all available data. The high admission counts in the year prior to study index highlight the extent of disease severity in both cohorts at baseline. This is also reflected in the high levels of breathlessness, airflow obstruction, and symptom burden within the RECEIVER cohort.

**Table 1:** Baseline Characteristics of the RECEIVER Trial Participants and the Location and Time-Period Matched Control Cohort

	RECEIVER	Control
**Number of individuals**	83	415
**Age at baseline, mean (SD), years**	64.4 (9.3)	64.6 (9.1)
**Sex, % female**	63.9	63.9
**COPD or respiratory-related admissions in previous year, mean (SD)**	2.46 (2.25)	2.47 (2.92)
**Smoking Status, %**		
**Former Smoker**	69.9	
**Current Smoker**	30.1	
**FEV1% Predicted, mean (SD)**	47.9 (20.8)	
**FEV1/FVC Predicted, mean (SD)**	0.46 (0.14)	
**Baseline CAT score, mean (SD)**	23.2 (6.6)	
**Baseline MRC Dyspnoea scale score, mean (SD)**^33^	3.67 (1.19)	
**Highest Eosinophil Count, mean (SD)**	0.64 (1.10)	
**Triple combination inhalers (LABA+LAMA+ ICS), % users**	80.72	
**Previous pulmonary rehabilitation, % had**	24.1	
**NIV therapy, % users**	28.9	
**Home oxygen therapy, % users**	37.3	
**Comorbidities, % with**		
**Osteoporosis**	13.3	
**Ischemic heart disease**	8.4	
**Obstructive sleep apnea**	12.0	
**Diabetes**	10.8	
**Asthma**	9.6	
**Atrial fibrillation**	9.6	
**Heart Failure**	10.8	
**Deep venous thrombosis/ pulmonary thromboembolism**	3.6	
**Cerebrovascular disease**	0.0	
**Bronchiectasis**	2.4	
**Pulmonary hypertension**	1.2	
**Pneumothorax**	2.4	
**Pulmonary fibrosis**	0.0	
**Lung cancer**	1.2	

**Notes**: Only age, sex, and admissions data are presented for the control cohort reflecting the data available for matching within the Safe Haven COPD dataset. Available data for the control cohort is shaded in blue.

**Abbreviation**: MRC, Medical Research Council.

Around 58% of the RECEIVER and 63% of the control cohort were resident in postcodes in the most socioeconomically deprived quintile of the Scottish Index of Multiple Deprivation (SIMD),^34^ a relative measure of socioeconomic deprivation across Scotland. The higher rates of COPD burden in lower SIMD quintiles within the RECEIVER and control cohorts are typical of COPD burden across NHS GG&C (Supplementary Table 2).

### Patient Application Utilization

**Figure 3:**
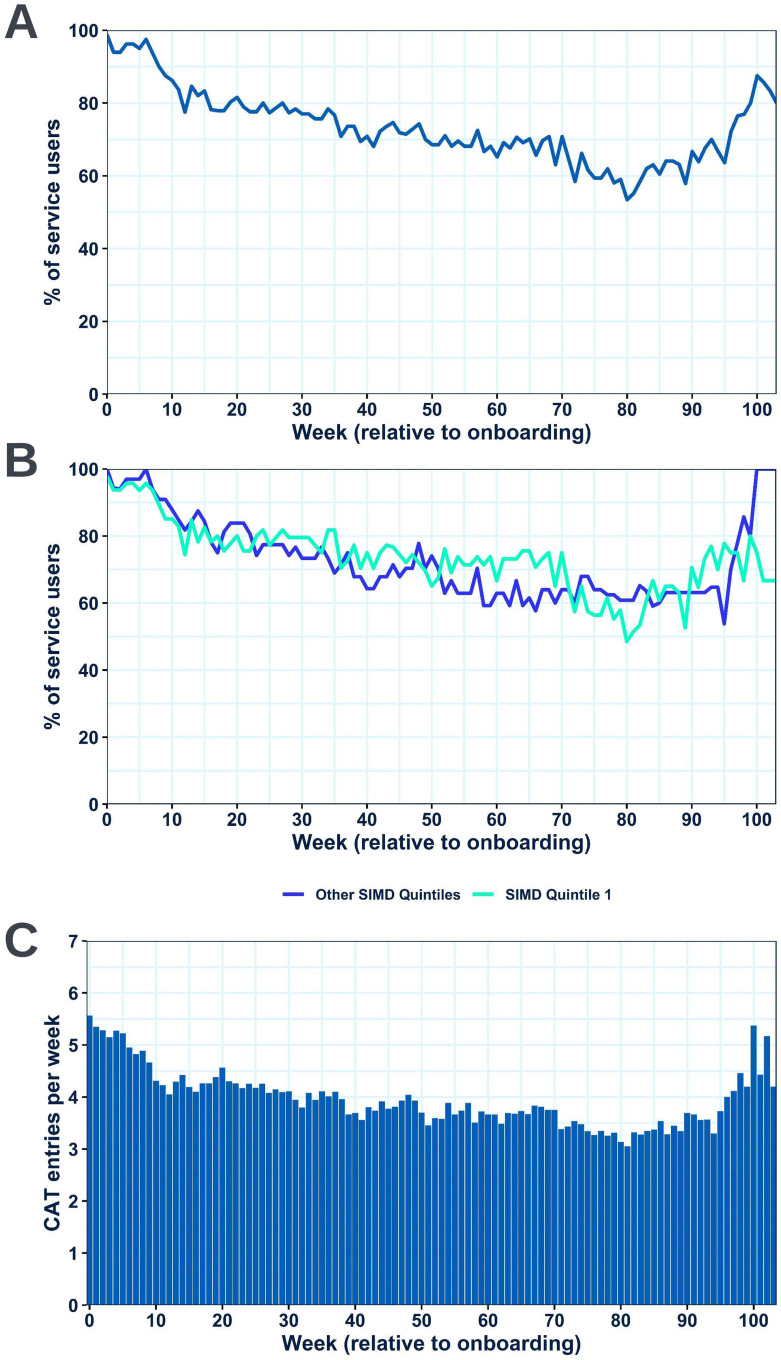
Utilization of the patient application as determined by completion of daily CAT entries. The percentage of participants completing at least one CAT entry per week is shown for the cohort overall (**A**) and the cohort stratified by SIMD quintile (SIMD quintile 1 vs SIMD quintiles 2–5) (**B**). The mean number of entries completed per participant per week is also shown (**C**).

The percentage of participants completing at least one CAT entry per week was ≥ 68.1% each week over the first year of follow-up (mean weekly completion 79.8%), with consistent usage noted beyond this (Figure 3A). Around 77% of participants were sustained users, completing at least one CAT entry per week on over 50% of possible follow-up weeks. Notably, 39 individuals in the cohort paused utilization for at least a week but 24 of them subsequently returned to regular or intermittent PRO completion. Analysis looking at utilization in participants resident in SIMD quintile 1 postcodes confirmed that utilization levels in this subgroup were comparable to utilization levels in the rest of the cohort (Figure 3B). Further analysis showed that the mean number of daily CAT entries completed per participant in the cohort overall across the length of follow-up was 4.0 (Figure 3C). Review of weekly MRC Dyspnoea scale and four-weekly EQ-5D VAS PRO question sets showed relative completion levels of these entries to be equivalent to CAT entry completion levels (Supplementary Figure 1).

**Figure 4:**
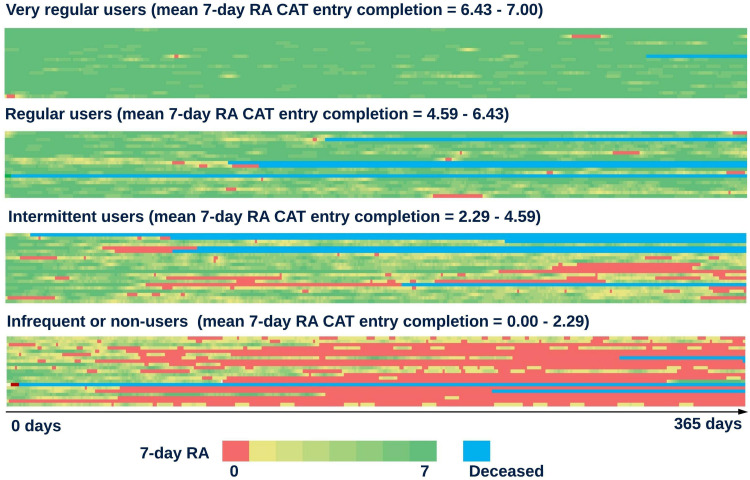
Heatmap visualization showing the seven-day rolling average CAT entry completion of each RECEIVER participant over the first year of follow-up, with the cohort stratified into quartiles based on this metric. Dark green areas represent periods of very high completion (6–7 daily CAT entries completed in the previous seven-days), whilst red areas represent periods of no recent completion (0 CAT entries completed in the previous seven-days). Continuous blue areas are shown where a participant has died.

**Abbreviation**: RA, rolling average.

Analysis characterizing utilization in more depth was also conducted by stratifying the cohort into four quartiles based on the mean number of entries completed in the previous seven days at each possible point over the first year of follow-up (seven-day rolling average completion) and conducting stratified analyses. A heatmap visualization displaying CAT completion over the first year of follow-up with the trial participants grouped into these quartiles is shown in Figure 4. Exploratory analysis found no significant differences in baseline characteristics and admission data in the year post-index date between the four utilization quartiles (Supplementary Tables 3 and 4).

### Survival Analyses: RECEIVER Vs Control

**Figure 5:**
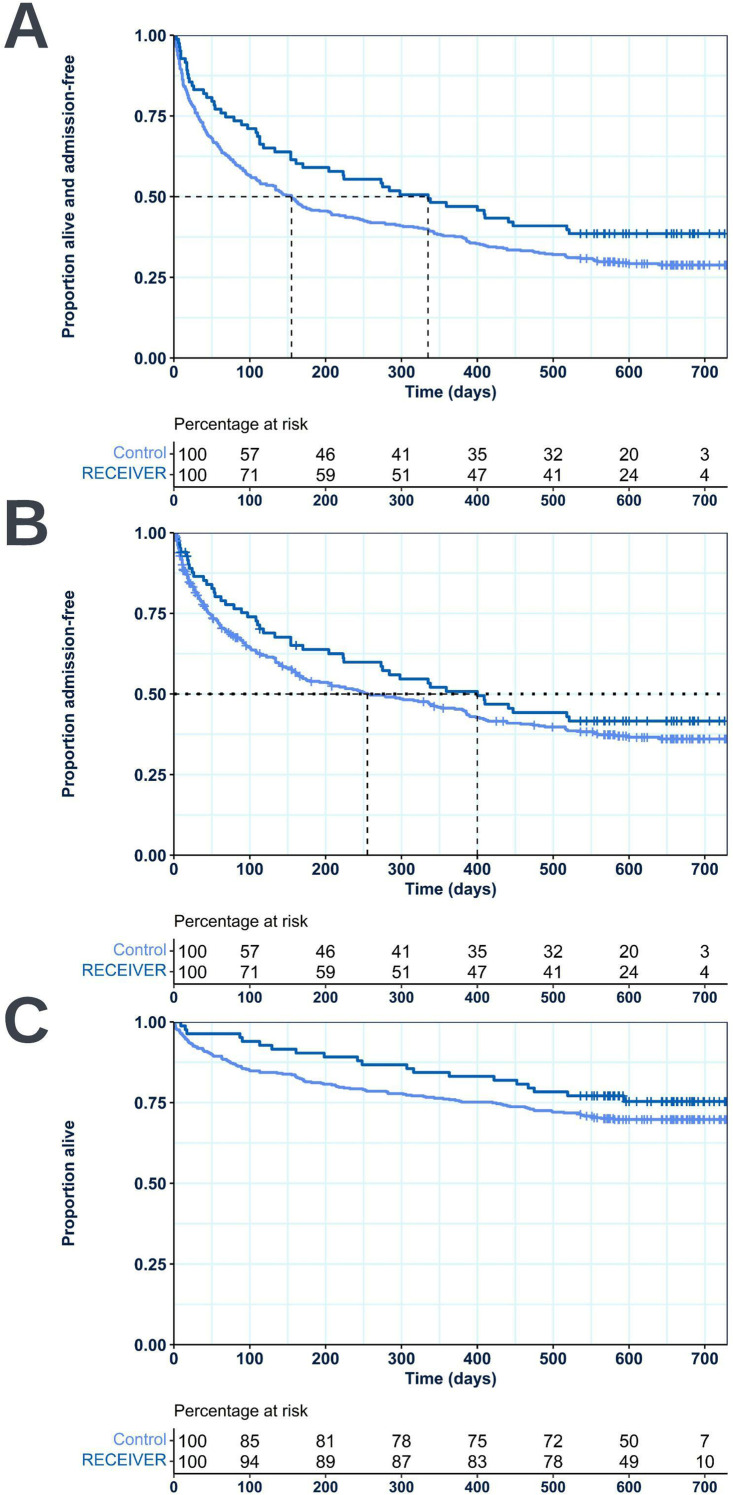
Survival plot visualizations with accompanying percentage at risk tables comparing survival to first COPD or respiratory-related admission or death (**A**), survival to first COPD or respiratory-related admission (**B**), and survival (**C**) from study index date between the RECEIVER cohort and the matched control cohort.

Median time to first COPD or respiratory-related admission or death was increased in the RECEIVER cohort compared to the control cohort (335 days vs 155 days) (Figure 5A). The difference in time to event across the follow-up period was significant between the cohorts (p = 0.047), with an unadjusted hazard ratio of 0.740 (0.550–0.996). A prolonged time to first COPD or respiratory-related admission was also noted in the RECEIVER cohort when considering this endpoint alone (400 days vs 255 days) (Figure 5B). However, the difference was not statistically significant between the cohorts (p = 0.241). 12-month mortality rate was also reduced in the RECEIVER cohort compared to the control cohort (16.9% vs 24.1%) (Figure 5C), however the difference in survival over follow-up was not statistically significant between the cohorts (p = 0.215). Survival data are detailed in Table 2.

**Table 2:** Table Comparing Survival to Event Metrics Between the RECEIVER Cohort and the Matched Control Cohort

	COPD or Respiratory-Related Admission or Death	COPD or Respiratory-Related Admission	Death
**RECEIVER (median time to event)**	335	400	n/a
**Control (median time to event)**	155	255	n/a
**Unadjusted hazard ratios (RECEIVER vs control)**	0.740 (0.550–0.996)	0.827 (0.603–1.135)	0.743 (0.463–1.191)
**Log rank test**	p = 0.047	p = 0.241	p = 0.215

**Notes**: Median time to event is shown for both time to COPD or respiratory-related admission or death and time to first COPD or respiratory-related admission. Unadjusted hazard ratios with corresponding 95% confidence intervals, and p-values for log rank tests which compare survival to each endpoint between the cohorts are also shown. Data for the control cohort is shaded in blue.

### Change in COPD or Respiratory-Related Hospital Admissions and Occupied Bed Days: RECEIVER Vs Control

**Figure 6:**
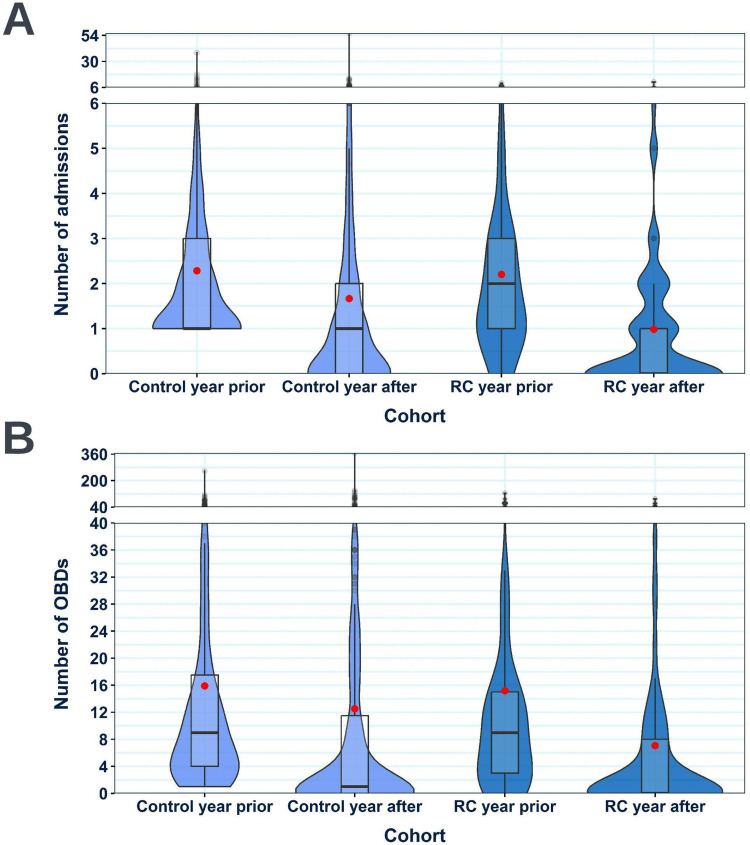
Violin boxplots comparing the number of COPD or respiratory-related admissions (**A**) and occupied bed days (**B**) had in the 12-month windows pre- and post-index date in the RECEIVER and control cohorts. Data shown is for individuals alive 12-months post-index date. Violin-box plots are selected to ensure complete data provision. For their interpretation: standard boxplots illustrate the variation of values (median and IQR), the relative frequency of individual data points is illustrated by the width of the violin plot at each point on the y-axis, and mean values are shown by red dots.

**Abbreviations**: OBDs, occupied bed days; RC, RECEIVER cohort.

The effect size of the reduction in annual COPD or respiratory-related admissions (Figure 6A) and occupied bed days (Figure 6B) was substantially higher in the RECEIVER cohort than in the matched control cohort in the year post-index date. Table 3 shows admissions data in the year windows pre- and post-index date for both cohorts. Separate analysis including all participants regardless of survival status in the follow-up year also showed greater reductions in the RECEIVER participants, despite shorter average follow-up time in the control cohort (Supplementary Table 5).

**Table 3:** Table Compiling Admissions Data for the RECEIVER and Control Cohorts

			Mean			Wilcoxon Signed-Rank Test Effect Size
			Year Before	Year After	Change	
**Admissions**	**Control**	n=315	2.29	1.67	0.62	0.423***
	**RECEIVER**	n=69	2.20	0.99	1.21	0.621***
**Occupied bed days**	**Control**	n=315	15.90	12.52	3.38	0.314***
	**RECEIVER**	n=69	15.19	7.07	8.12	0.535***

**Notes**: Mean COPD or respiratory-related admission and occupied bed day counts in the 12-month windows pre- and post-index date are shown. The mean change for each cohort and the effect sizes of these changes (Wilcoxon signed-rank test) are also displayed. Data shown is for individuals alive 12-months post-index. ***p < 0.001, Wilcoxon signed-rank test. Data for the control cohort is shaded in blue.

### Health-Related Quality of Life and Symptom Burden Over the Study Period

**Figure 7:**
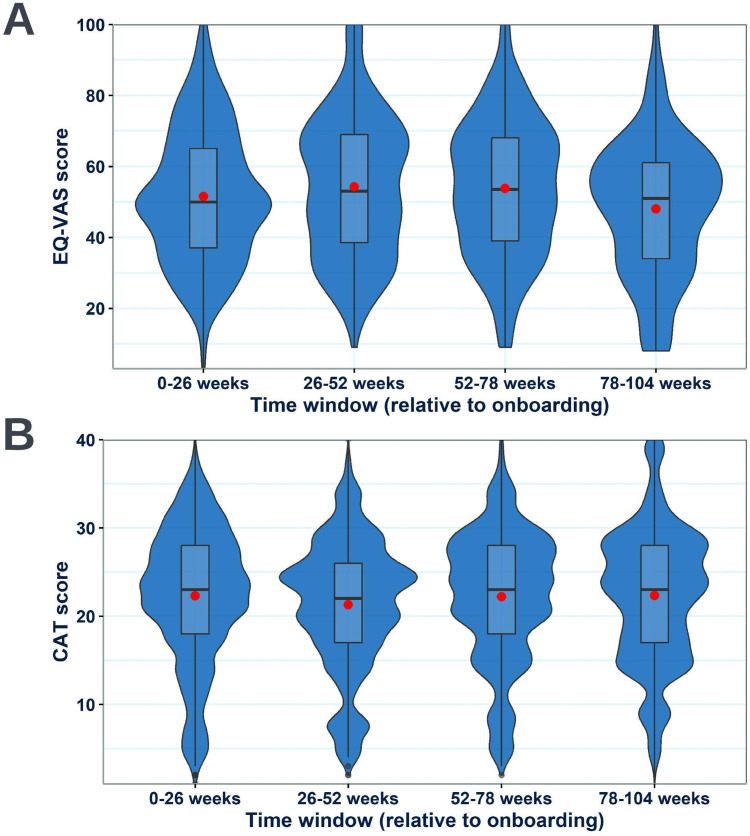
Annotated violin boxplots showing the change in the distribution of EQ-VAS (**A**) and CAT scores (**B**) reported by the RECEIVER participants across 26-week windows relative to onboarding. Violin-box plots are selected to ensure complete data provision. For their interpretation: standard boxplots illustrate the variation of values (median and IQR), the relative frequency of individual data points is illustrated by the width of the violin plot at each point on the y-axis, and mean values are shown by red dots.

Health-related quality of life and symptom burden data were obtained throughout the study period through the once four-weekly EQ-5D-visual analogue scale (EQ-VAS) and the once daily COPD assessment test (CAT) entries, respectively. Changes in the distribution of reported values for these metrics over four equal time windows in the trial relative to onboarding were investigated. Health-related quality of life (Figure 7A) and symptom burden (Figure 7B) were both shown to be broadly stable over the course of the study.

### Community-Managed Exacerbation Count Recorded Through the Patient Application

**Figure 8:**
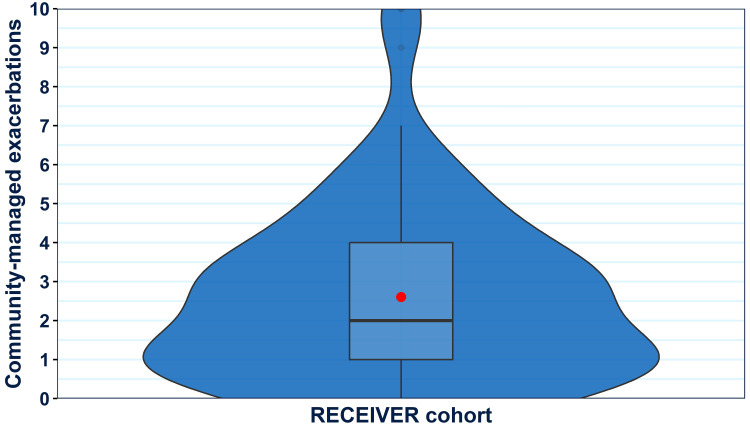
Violin boxplot showing the distribution of participant-reported community-managed exacerbation counts within the RECEIVER cohort during the first year of follow-up. A violin-box plot is selected to ensure complete data provision. For interpretation: a standard boxplot illustrates the variation of values (median and IQR), the relative frequency of individual data points is illustrated by the width of the violin plot at each point on the y-axis, and the mean value is shown by a red dot.

RECEIVER participants reported a median of 2 community-managed exacerbations per year in the 12-months post-index (Figure 8). A higher median of 4 participant-reported community-managed exacerbations was noted in the quartile of RECEIVER participants with highest patient application utilization (Supplementary Figure 2).

## Discussion

The objectives of this study were to determine the acceptability, feasibility, and utility of deploying the co-developed COPD digital service alongside routine clinical care for people with severe COPD, as well as to further develop the evidence base for digital self-management and remote monitoring interventions in the management of COPD. Focus was placed on determining service utilization over a prolonged period, and evaluating the impact of service adoption on clinical outcomes. Sustained and high levels of interaction with the patient application were illustrated in the trial, including in participants resident in postcode areas in the most socioeconomically deprived quintile of the SIMD. Improved survival metrics and admission reductions were also noted amongst the participants in comparison to a location and time-period matched control cohort with a comparable admissions profile in the year prior to study index date, as well as an equivalent age and sex distribution. These outcomes were obtained with a median of 2 community-managed exacerbations recorded per participant per year, and broadly stable quality of life and disease impact metrics recorded across the study follow-up period, despite the progressive nature of COPD.

Some major strengths of the study were the detailed characterization of patient application usage and incorporating study follow-up beyond one year. It was very encouraging that most participants continued to complete at least one CAT entry per week across follow-up and that even participants with interrupted usage generally resumed interactions at a later point. The average completion of four CAT entries per participant per week throughout the study period was also very encouraging, with a notable proportion of participants sustaining daily usage. Within the RECEIVER trial, participant symptom data was reviewed only on an as required basis (alongside scheduled care reviews or in response to patient-initiated unscheduled contact). This detailed symptom trend and insight data were available to enrich these reviews, reducing patient-clinician time spent on information gathering, without increasing clinical team workload by mandating regular reviews of the PRO data. It will be valuable to explore in subsequent service evaluations which components of the digital service are most useful to patients and clinicians in different scenarios.

In addition to providing the clinical team with granular data for clinical decision support, this data will be highly valuable for ongoing machine learning analysis to generate predictive models that could potentially improve COPD management further. Exploratory analysis to determine if any demographic factors, factors related to disease severity, comorbidities, additional therapies or admission rate over follow-up were associated with utilization, did not identify any significant differences in these metrics between participants with different application utilization frequencies. The difficulty in identifying discrete characteristics associated with utilization of digital interventions has been widely reported elsewhere.^35^ This indicates that there is not an obvious subpopulation of people with COPD who should be targeted for selective provision of digital tools.

Out of a potential 283 RECEIVER participants, only 41 lacked technology access. This was lower than anticipated and is an improvement when compared to previous reports.^36^ However, efforts should be made to investigate potential solutions to address this barrier in access. The increase in technology access could be partly due to rapidly growing internet usage amongst older adults in the UK. Recent survey data showed that the proportion of people over 75 who were recent internet users nearly doubled between 2013 and 2020.^37^

COPD prevalence is disproportionately high in more deprived communities and deprivation is independently associated with increased risk of COPD hospitalization and mortality.^2^ It was therefore important to evaluate the trial participant population in terms of SIMD distribution, especially considering the association of deprivation with digital exclusion and unequal access to care in more socioeconomically deprived communities across the UK.^38^ Reassuringly, when comparisons were made between utilization levels amongst the sub-cohort of participants resident in the most deprived SIMD quintile and participants in other SIMD quintiles, equivalent utilization levels were seen (see the Supplementary Material for a breakdown of the RECEIVER and control cohorts by SIMD quintile).

The improvements in survival metrics and admission reductions compared to the control cohort provide reassurance about the safety of digital co-management of COPD, when it is co-designed and deployed as part of transformation of routine care. Analysis of qualitative data on the patient-perceived benefits of the COPD digital service is in progress, and this should provide insight into the potential mechanisms by which the benefits observed here have been realized. It is our anticipation that the digital tools help support guideline-based care including COPD self-management. The improved participant outcomes seen in the RECEIVER cohort compared to the control cohort are in line with those seen in participants who were successfully taught self-management in a previous trial undertaken in our organization, supporting this view.^39^

Interpretation of these clinical outcomes should be measured: this was an observational study with risk of multiple biases which can only be partly mitigated by the use of a location and time-period matched routine clinical dataset derived control cohort. It is notable that reduced hospital admission rates were seen in the control cohort in the year following study index. This effect has been widely reported over the period overlapping with COVID-19 restrictions and is attributed to a reduction in community transmission of respiratory infections that can trigger COPD exacerbations.^40^ This indicates that the control cohort is representative of the broader population with severe COPD and that it is likely to be a suitable comparator for the RECEIVER cohort which establishes a baseline COVID-19 impact on outcomes in the absence of the digital COPD service. The lack of a traditional randomized control arm and the incomplete information for the control cohort are important caveats. However, accepting lack of availability of desirable information including smoking rates and lung function data is a necessary compromise when using routine clinical data for control analyses such as these. Additionally, the control cohort derivation from the same health board as the trial participants and the matching of the index date and outcome follow-up periods to the RECEIVER participants reduces potential bias from seasonality, location, and COVID-19 pandemic impacts on COPD event rates. This study design also allowed for participant numbers using the intervention to be maximized for the interim and final primary endpoint analyses. The biases and compromises inherent in the use of routine clinical data are mitigated as far as is able, and our conclusions are tempered accordingly.

With all of this noted, the observed improved outcomes amongst RECEIVER participants are encouraging both at an individual and population level. In addition to improving outcomes for people with COPD, the observed reductions in COPD or respiratory-related occupied bed days observed amongst the RECEIVER participants could have wider benefits to health-care systems, such as cost savings resulting from avoided hospitalizations and alleviating strains on hospital capacity. Additionally, reductions in admissions reduce the environmental impact of COPD management per individual per year, with hospital admissions accounting for a considerable proportion of NHS emissions.^41^ This has been under focus recently in the UK, with the NHS plan for reaching net zero emphasizing the importance of moving towards an out of hospital preventative model of care to improve outcomes and reduce emissions simultaneously.^41^

The findings in the study are consistent with several previous studies on telehealth, remote-monitoring, and supported self-management-based COPD digital services that showed improved clinical outcomes.^16^,^17^ Conversely, they are inconsistent with studies that did not show improvements in clinical outcomes with these types of services.^42^,^43^ There were several key differentiators of the evaluated COPD digital service compared to those described in other studies. These include patient and clinician co-design of the intervention to maximize usability, accessibility, and utility of the intervention, verified COPD diagnosis at onboarding, daily prompts to complete PROs, the opportunity for asynchronous patient-clinician messaging, clinician option to add individualized inhaler prescription and rescue pack information in the patient application, and use of the service alongside routine care contacts rather than at prespecified regular data reviews.

Based on these evaluations, scale up of the digital support service to other health boards has progressed and some of the key results of the trial have been summarized in a plain language flyer to encourage additional individuals with COPD within the NHS GG&C health board to sign up to the support service (Supplementary Figure 3). Some of the next steps include analysis of patient application utilization and clinical outcomes amongst the individuals who were onboarded as part of the COVID response, as well as analysis of RECEIVER participant physiology and therapy data from wearables and home NIV machines. Implementation of AI-based actionable insights is in development, with ongoing work to operationalize machine-learning models trained and validated using data collected during the RECEIVER trial and Safe Haven data.

## Conclusion

The primary objective of the RECEIVER trial has been successfully delivered, with sustained participant usage of the co-designed digital service confirmed. An improvement in clinical outcomes in the trial participants compared to a matched control cohort derived from routine clinical data was also observed. Based on these positive findings, scale up of service provision to support end-end proactive and preventative COPD care is in progress. The rich user experience and structured datasets collated in the RECEIVER trial will support the development and deployment of future digital innovations, including the use of AI derived insights, to further enhance care and continue the drive to improve outcomes for people living with COPD.

## Data Sharing Statement

The data that support the findings of this study are available for access via NHS GG&C Safe Haven. Applications for de-identified data access would be reviewed by the NHS GG&C Local Privacy and Advisory Committee and might incur data access fees.
